# Mental Health Treatment Needs and Preferences for People Living with Bipolar Disorder in Australia

**DOI:** 10.1007/s10597-025-01495-4

**Published:** 2025-08-01

**Authors:** Chelsea Ho, Eileen McDonald, Tania Perich

**Affiliations:** 1 School of Psychology, Western Sydney University, Locked Bag 1797, Penrith, NSW 2751 Australia; 2 Bipolar Australia, Sydney, Australia; 3 Translational Health Research Institute, Western Sydney University, Penrith, Australia

**Keywords:** Bipolar disorder, Treatment needs, Mental health, Service delivery, Australia

## Abstract

People living with bipolar disorders may face a range of treatment challenges, however, the treatment needs of those living with bipolar disorder in Australia have not been directly assessed. The present study aimed to explore the treatment and care preferences of people living with bipolar disorder in Australia. Participants were part of a large co-designed survey that assessed preferred settings, barriers, and access to treatment. A total of 494 participants provided responses regarding preferred treatment settings with 188 (38%) preferring the public system, 175 (35%) private, and 153 (31%) indicating a preference for both/either private or public care. The setting that was most frequently endorsed was at home (*n* = 343; 69%), then outpatient (*n* = 155; 31%), and inpatient (*n* = 93; 19%). Affordability, resourcing, geographical and timely access, improving education and addressing stigma were reported as key unmet needs, indicating that more work is needed to improve access to care for Australians.

## Introduction

Bipolar disorder is a chronic mood disorder characterised by mood episodes that swing from extreme highs (i.e. mania or hypomania) and extreme lows (i.e. depression) (American Psychiatric Association [APA], 2022). In Australia, the 12-month prevalence of bipolar disorder from 2020 to 2021 was 2.2% (Australian Bureau of Statistics, 2023). The age of onset for bipolar disorder is usually between the early- to mid-20s and the first episode of illness is typically depressive (APA, 2022). Many individuals experience a delay of diagnosis by 10 years after symptom onset due to missed diagnoses from clinicians failing to properly assess symptoms and the rarity of individuals making spontaneous reports of hypomanic symptoms (Di Salvo et al., 2023; Goes, 2023). Inaccurate and delayed diagnoses have severe implications, including elevated rates of suicide attempts and medical comorbidities, and higher direct and indirect medical costs (Di Salvo et al., 2023).

Bipolar I disorder is defined by the presence of at least one lifetime manic episode where there is elevated, irritable, or expansive mood, and a significant increase in energy levels for at least 1 week (APA, 2022). In contrast, a diagnosis of bipolar II disorder requires the occurrence of at least 1 lifetime hypomanic episode and at least 1 lifetime depressive episode (APA, 2022). Differences are noted between bipolar I and II in a range of areas including illness course features (e.g. greater time depressed for bipolar II) and functioning status, with bipolar II disorder having higher socioeconomic status compared to bipolar I disorder (Tondo et al., 2022). Use of medication and treatment approaches also differ between subtypes, due to the nature of symptoms reported (e.g. fewer psychotic symptoms for Bipolar II diosrder) and their corresponding treatment needs (Tondo et al., 2022).

Recovery models have been widely adopted globally into standard mental health treatment and care. Recovery-orientated approaches focus on personal recovery, rather than symptomatic recovery. They highlight the need for personal well-being and aim to move away from a negative personal identity associated with mental illness (Dell et al., 2021). Aspects of recovery may further include a focus on improving social and environmental conditions, access to resources, autotomy and personal responsibility (Dell et al., 2021).

Recovery orientated approaches often integrate peer perspectives, such as through peer support services, enhance engagement with services and also increase consumer choice in treatment and care (Chatwiriyaphong et al., 2024). They may further include the addition of specific strategies, such as assistance with housing and employment support. Clinicians working within recovery frameworks perceive them to be positive, although barriers such as uncertainty regarding the concept of recovery have been noted (Chatwiriyaphong et al., 2024). These models are currently employed in Australia across a range of mental health care settings (e.g. NSW Health, 2025), however many have previously reported a need for more focus on recovery-orientated services, with this approach not being implemented in many settings (State of Victoria, 2021).

Despite recovery-orientated approaches showing promise, international studies on those indicate that treatment needs and preferences are not being fully met by current health care practices. For example, a UK study of 28 participants, 5 diagnosed with bipolar II disorder, noted several unmet needs during clinician interactions and informational needs (Bilderbeck et al., 2014). Participants described lack of discussion of illness symptoms, causative factors for illness, available treatment options, strategies for self-management, and nature of ongoing support following diagnosis of bipolar disorder.

In addition, a large web-based survey of adults living in the United States with self-identified depression and/or bipolar disorder (*n* = 6153) found that over 90% of respondents wanted better treatment and care (Morton et al., 2022). Approximately 75% of participants felt that their treating team were focused on symptom minimisation and 38% of participants were unsatisfied that their treating team only focused on one issue at a time. Focus groups revealed that patients had a broader conceptualisation of wellness than clinicians as comprising both mental and physical health (Morton et al., 2022).

In a qualitative Australian study exploring factors which influence treatment decision- making of people living with bipolar II disorder (*n* = 28), unmet care needs in terms of professional support were also reported (Fisher et al., 2018). Semi-structured interviews revealed that participants valued continuity of care and a therapeutic alliance characterised by trust and respect for patient preferences. Where these needs were unmet, patients reported feeling unsupported, lacked satisfaction with care, and lacked confidence in managing symptoms and adhering to treatment.

Clinician knowledge about treatment options and providing easily understandable information were also common unmet needs, impacting trust in the clinician and undermining treatment adherence (Fisher et al., 2018). Additionally, despite general preference for patient- or family- led decision making or shared decision-making, many patients and family members reported experiencing clinician-led decision-making which stripped participants of perceived autonomy and agency. Longer consultations with general practitioners (GPs) and psychiatrists and booking follow-up consultations were identified as promoting treatment adherence and facilitating better decision making. Participants also identified high costs of accessing psychiatrists and psychologists, living outside metropolitan areas, and inadequate government subsidisation as systemic barriers to better care. This is consistent with other studies where it has been found that those who have been diagnosed with a mental health condition were 7.65 times more likely than people who did not have any health conditions to forgo treatment due to cost (Callander et al., 2017).

In circumstances where consumers may not be limited by geographical or economic barriers, the complexity and severity of the condition plays a role in determining the treatment setting (Malhi et al., 2021). In addition, although the existence of the public and private systems is aimed at providing consumers with more choice and improved access, access is mediated by a range of other factors such as economic and geographical factors (Malhi et al., 2021). Thus, although preferences may be noted on the part of the individual, they may not necessarily be able to engage preferred forms of treatment due to these external factors.

In terms of preferences for early interventions services in Australia, previous research in a small sample (*n* = 82) noted that participants preferred BD-specific early intervention services, with face-to-face delivery being preferred over online options (Gates et al., 2024). In line with recovery-orientated approaches, participants endorsed integrated peer support as being a preferred aspect of early intervention services, suggesting that this is an important component of early intervention treatment and care. However, the study did not assess private or public treatment preferences, nor treatment preferences for other mental health services.

Broadly, Australian data suggests that the Australian mental health system is facing a range of challenges, with many reporting difficulties with access and cost, noting poor staffing and confirming that obtaining treatment and care is difficult (State of Victoria, 2021). However, the treatment needs of those living with bipolar disorder in Australia specifically have not been assessed and this is largely unknown.

Recovery-orientated models indicate the need to integrate patient preferences into treatment delivery and care. In line with this approach, this present study aimed to assess treatment preferences for those living with bipolar disorder in Australia, in a study that was led by consumers Specifically the study aimed to assess treatment regarding preferences for healthcare system engagement (public vs. private care) key barriers to receiving treatment and care. Government support required, crisis care, inpatient care, and access to mental health care were also investigated. The questions were developed by consumers and addressed areas identified by members as being important to assess in relation to the provision of mental health services for those living with bipolar disorder in Australia. The study further aimed to assess key demographic variables that may be associated with responses.

## Method

### Participants

Participants were part of a larger study that aimed to investigate the needs of people living with bipolar disorder in Australia. Participants were recruited via Bipolar Australia, Facebook and Instagram, and flyers were distributed in GP clinics, mental health and psychology practices across Australia in all States and Territories. Eligibility criteria required participants to be over 18 years of age, speak English, have a diagnosis of bipolar disorder (given previously to the participant by a healthcare practitioner), and live in Australia. Participants self-selected to take part. Informed written consent was obtained from all participants. The study was approved by the Western Sydney University Human Research Ethics Committee (H15234).

A total of 566 participants (443 female [78%], 107 [19%] male, 16 [3%] other) took part in the survey with a subsample of 494 participants providing a response to any of the questions that were asked in the section regarding treatment setting needs and ways the government could improve access to treatment and care. Age ranged from 18 to 82 years, with a mean age reported of 46.75 (SD = 13.76). Key sample characteristics are presented in Table 1.

**Table 1:** Demographic characteristics

Demographic details		*N* = 566	%
Marital status			
	Married/Defacto	255	45
	Separated, divorced or widowed	147	26
	Never married	164	28.9
Do you currently have a job?			
	Yes	313	55.3
	No	253	44.6
If yes, what type of job is it?			
	Full time	135	23.8
	Part time	100	17.6
	Casual	78	13.8
Are you currently receiving government benefits?			
	Yes	255	45.1
	No	311	54.9
If yes, which type?			
	Unemployment benefit	54	9.5
	Disability Support Pension	136	24
	Carer’s Pension	10	1.8
	Other (Please state)	55	9.7
What is your highest level of education?			
	Did not attend school/attended primary school	3	0.5
	Some secondary (Year 10)	63	11.1
	Completed secondary (Year 12)	53	9.3
	Vocational qualification, diploma or associate diploma	150	26.5
	Bachelor or higher degree	266	47
	Other	31	5.5
State	ACT	13	2.3
	NSW	270	47.8
	QLD	85	15
	SA	51	9
	TAS	18	3.2
	VIC	78	13.8
	WA	46	8.1
	NT	5	0.9
Region type	Metropolitan zone (RRMA 1 and 2)	391	69
	Rural zone (RRMA 3 to 5)	163	28.8
	Remote zone (RRMA 6 and 7).	5	0.9
	Unsure	5	0.9

### Procedure

Participants who were recruited via social media accessed the survey via a web link. Flyers contained a QR code for respondents to scan via their mobile phone to access the survey. Participants were instructed to review the participant information sheet, provide written consent, and complete eligibility questions before providing demographic details and completing the questions investigating their unmet needs. Participants were not required to answer all survey items. Participants were provided with the telephone number to Lifeline and encouraged to access this service in the event of distress. The survey was hosted on Qualtrics. The questions were co-designed with members of Bipolar Australia, described as the peak national, not-for-profit organisation, supporting people living with bipolar disorders, families and carers, mental health services and professionals. The study aims and scope was defined by Bipolar Australia, who approached the researchers with overall scope of the survey in the first instance. Specific areas were then proposed by Bipolar Australia and reviewed by the key members. Questions were developed by the researchers and then sent to Bipolar Australia for further review and refinement prior to submission to the ethics committee.

### Materials

The questions covered domains such as demographic questions, history of diagnosis, and current and past use of psychiatric care. Participants were asked ‘Would you prefer to access support, treatment and care through the public or private system?’ and were given the option of ‘Public’, ‘Private’, or ‘Both’. Follow up questions included ‘Where would you prefer to get early support for your mental health if you needed care?’. Participants were then asked to select options either ‘At Home’, ‘Half-way house with peer support’, ‘In hospital’, ‘Outpatient in public system’, ‘Other’.

Additional open-ended questions were: “What more could the government do to help improve access to mental health care for people living with bipolar disorder?”, “What more could the government do to help improve Medicare for people living with bipolar disorder?”, “What are the key barriers that you see that prevent people living with bipolar disorder from getting the best possible treatment and care in Australia?”, “What are the key things that could be done that would help people living with bipolar disorder get best possible treatment and care?”, “What can be done to improve immediate help available to people in crisis?”, “What should inpatient mental healthcare look like in 10 years’ time for people living with bipolar disorder?”, and “What needs to change in order to realise that vision?”.

Participants were also asked to indicate on a scale from 1 to 10 ‘How satisfied are you with the most recent treatment you received in the private system?’ and ‘How satisfied are you with the most recent treatment you received in the public system?’.

### Data Cleaning

Data was cleaned and prepared for quantitative analysis by transforming string data into numerical data and ensuring consistent naming conventions. System missing data points were coded as missing.

### Data Analysis

Responses to open-ended questions were exported into Excel and analysed inductively using conventional content analysis. This approach was selected because it is appropriate for areas of research where existing theory or literature is limited (Hsieh & Shannon, 2005). Inductive conventional content analysis systematically classifies large amounts of text-based data into content categories and allows the frequency of responses under each category to be quantified (Tesch, 2013). For each question, all responses were read for data immersion and to form an overall impression.

In line with the inductive approach, author CH generated initial codes for each response and then conceptually related codes were collated into themes. The organisation and definitions of sub-themes and themes were reviewed next, and some sub-themes were combined during this process. The coding framework was confirmed following discussion between the authors. Themes were then consolidated and coded with a research assistant and cross checked and reviewed.

Frequencies for each theme and sub-theme were then coded in IBM SPSS Statistics to facilitate quantitative analyses. All quantitative analyses were conducted using IBM SPSS Statistics. Results of the content analyses are reported as a quantitative summary of the themes and sub-themes, and as a qualitative description of the thematic and sub-thematic findings. Quantitative findings are reported as descriptive statistics.

### Planned Analyses

A series of two-way chi-square analyses were conducted to test whether there were any socio-demographic variables associated with preferences for engaging with the public and private health systems, and preferences for care settings.

Socio-demographic variables included are gender (male/female), relationship status (married or de facto/separated, divorced, widowed or never married), born in Australia (yes/no), bipolar type (bipolar I/bipolar II), employment status (unemployed/employed), highest level of education attained (secondary or less; vocational training or others/completing or completed tertiary), whether in receipt of government benefits (yes/no), metropolitan/rural or remote area of residence (yes/no) and whether there was a lifetime diagnosis of another mental health problem (yes/no).

Independent samples t-tests were planned to investigate whether preferences for engaging with the public and private health systems or preferences for care settings (independent categorical variables) differed by age (dependent continuous variable). Age analyses were not conducted for responses for peer supports, alternative settings and half-way housing due to small numbers of participants endorsing these responses.

## Results

A total of 494 participants provided responses for questions regarding preferred treatment. A total of 188 (38%) participants preferred public health, 175 (35%) preferred private health, and 153 (31%) indicated a preference for both/either private or public care. See Table 2 for significant chi square demographic features associated with public/private system treatment preferences.

**Table 2:** Socio-demographic variables associated with treatment preferences

Preference		Demographic feature	*N* (%)	Odds ratio	95% Confidence interval		χ2	*p*-value
					Lower	Upper		
Public or Private preference	Public	Located in rural or remote area	65 (46%)	1.195	1.01	1.415	4.866	0.027
		Part-time, casual or unpaid work	70 (44%)	1.378	1.163	1.632	13.307	*p* <.001
		Currently receiving govt benefits	105 (56%)	1.589	1.266	1.993	16.318	*p* <.001
		Did not obtain university degree	111 (44%)	1.351	1.072	1.702	6.697	0.01
	Private	Located in metropolitan area	137 (40%)	1.637	1.194	2.244	10.699	0.001
		Obtained university degree	96 (40%)	1.154	1.01	1.317	4.552	0.033
	Both	Employed full time	52 (56%)	1.709	1.226	2.384	10.151	0.001
Setting preference								
	Not at home	Did not obtain university degree	91 (36%)	1.422	1.081	1.87	6.509	0.011
	Hospital	Married or in a defacto relationship	51 (23%)	1.451	1.005	2.098	3.898	0.046
	Not outpatient in public health	Bipolar I disorder	52 (26%)	1.188	1.047	1.348	6.954	0.008
	Peer support (e.g. safe haven hubs)	Not married or in a defacto relationship	41 (15%)	1.085	1.018	1.157	6.109	0.013
	Half-way housing with peer support	Receiving govt benefits	18 (8%)	2.26	1.065	4.796	4.789	0.029
	Other	Not employed	24 (11%)	1.068	1.011	1.127	6.181	0.013

The setting that was most frequently endorsed for early mental health support was at home (*n* = 343; 69%), followed by outpatient (*n* = 155; 31%), then inpatient (93; 19%), peer supports (*n* = 59; 12%), alternative settings (*n* = 38; 8%) and half-way housing (*n* = 28, 6%). Participants who preferred an at-home setting were significantly younger (M = 45.64, SD = 12.901) than those who did not (M = 48.89, SD = 15.635), t=−2.415, *p* =.016. Those who preferred an outpatient setting were also younger (M = 43.15, SD = 13.262) than those who did not endorse this (M = 48.23, SD = 13.854), t=−3.826, *p* <.001. See Table 2 for significant chi square demographic features associated with treatment preferences.

For those who chose public care, cost was the most common reason (*n* = 126, 67%) and not having access to private health care cover was the second highest endorsed response (*n* = 62, 33%). For those who chose private care, access to better or more appropriate care was the most endorsed type of response (*n* = 53, 30%). Experiences in public care was the second highest endorsed response (49, 28%). For those who chose both public and private, the most endorsed reason was cost (39, 26%) followed by better or more appropriate care (37, 24%).

For the set of questions relating to improving access to treatment and care overall, the most endorsed response was to reduce the costs associated with managing bipolar disorder (*n* = 179, 38%) followed by increase resources (funding, services and infrastructure) (*n* = 178, 37%). Increasing staff and reducing wait times was the next most endorsed response (*n* = 103, 22%). For Medicare, the most endorsed response was reducing the gap for psychiatry (*n* = 179, 38%), followed by providing more psychology sessions (*n* = 149, 34%) and increase bulk billing (*n* = 83, 18%). See Table 3 for example quotes from participants.

**Table 3:** Quotes from participants by theme for ways the government can help improve access to mental health care and medicare

Theme	Examples
Ways the government can help improve access	“More Better Access psychology appointments per year… Recognise that it’s ridiculous to attempt to treat a severe chronic illness in ten sessions.”
	“Bulk billed GP telehealth for people with complex mental health conditions. It worries me to think with less bulk billing people may delay getting a script and accessing medication.”
	“More awareness and education for communities.”
	“Reduce wait times for appointments.”
	“More access to mental health outside the major cities. There is NOTHING in rural or remote areas.”
Improve Medicare	“Make mental health care FREE or extremely reduced in cost.”
	“Increase the Medicare rebates for GP (especially long appointments) and psychiatrists. Increase the Medicare rebate and number of appointments for psychology.”

A total of 99 (23%) participants reported that more alternative care settings are needed for crisis care, such as safe haven options. Participants also reported a need for more resources such as staff, beds and funding (*n* = 82, 19%), more telephone or external support (*n* = 57, 13%), and improved access, reduced wait times and receiving more immediate care (*n* = 46, 11%). See Table 4 for example quotes from participants.

**Table 4:** Example quotes from participants regarding barriers and improve crisis and inpatient care

Theme	Examples
Key Barriers	“Access to and affordability of required services. Psychiatric, psychological, and counselling services should be more readily available.”
	“Time availability as not many practitioners conduct medicinal visits out of office hours.”
	“I think cost of treatment is a large factor. Between psychologist, psychiatrist, medication, and other expenses it is very expensive”
	“Lack of educational training for people living with bipolar to learn more about management.”
Removing barriers	“Better voluntary access to residential/inpatient stays.”
	“Telehealth seems to work well giving better access.”
	“Further education for GPs and psychologists who are likely to be the first people someone with undiagnosed bipolar will present to.”
	“Providing services. Outpatient courses in public hospitals similar to private hospitals.”
Improve crisis care	“Improve waiting times in public EDs.”
	“Don’t refuse care to people or turn them away (people die because denied or left waiting for hours and hours).”
	“More specialised mental health professionals to assess and recommend treatment options at Emergency Departments or specialist clinics. Emergency room doctors and nurses are simply too busy to adequately assist people in the midst of a mental health crisis.”
Improve inpatient care	“The design of mental health inpatient facilities that look and feel less like a punishment facility.” “Reduce restrictive practice. Not restrain people.”
	“Less need for ED access process to psych ward.”
	“Better education of medical and policing staff to ensure that people are not mistreated when in distress.”
	“Removing stigma of being in a ‘psychiatric hospital.‘”

In response to the question, ‘What should inpatient mental healthcare look like in 10 years’ time for people living with bipolar disorder?’, a total of 149 (36%) participants indicated they would like to see a change in the support levels, 110 (27%) participants indicated they would request a change in physical environment, 99 (24%) participants said they would like better communication and 89 (22%) participants reported they would like more therapies offered.

In response to the question ‘What needs to change in order to realise that vision?’, 160 (39%) participants said more education, training or less stigma, 148 (36%) participants said more funding, 119 (30%) participants said more government support and 68 (16%) participants indicated a change in attitudes towards mental health, or that the whole system needed to change.

### Barriers To Care

The most common response was financial (*n* = 229, 52%) with 163 (37%) participants noting availability and access to services as a significant concern. Other commonly endorsed responses included stigma and awareness of the condition (*n* = 160, 36%) and personal factors relating to the illness itself (e.g., mania symptoms) (*n* = 41, 9.3%). Misdiagnosis was listed by 31 (7%) participants. The most common suggestions to improve care included the provision of more services broadly in both community and hospital settings (*n* = 220, 50%), removing financial barriers (127, 29%), and improving understanding and increasing staff training (*n* = 127, 29%). See Table 4 for example quotes from participants.

### Satisfaction Scores

A total of 415 participants answered the question regarding ‘How satisfied are you with the most recent treatment you received in the public system?’ with a mean of 5.26, SD of 3.24, and range 1 to 10 being reported. A total of 333 participants answered the question ‘How satisfied are you with the most recent treatment you received in the private system?’ with a mean of 6.93, SD of 3.03, and range 1 to 10 being reported.

## Discussion

The present study describes results from the largest study that has investigated the treatment and care preferences and needs of people living with bipolar disorder in Australia. The public and private health systems were equally favoured in the sample and the majority of participants reported that the home environment was the more preferred setting for early intervention and care. This is the first study to report on these findings in Australia, given that prior research did not assess location preferences or sector preferences in detail (Gates et al., 2024).

The cost of treatment and care was the most recognised barrier to accessing mental healthcare for participants. This is consistent with prior government reports noting access and cost barriers to care in Australia for those living with a mental health condition (State of Victoria, 2021) and suggests that this is a key barrier for those living with bipolar disorder specifically. Many respondents also stated that the government can improve Medicare by directly increasing Medicare funding to provide more services and financial support. Government policy should consider adopting these recommendations to ensure that treatment needs are met for those who obtain outpatient treatment outside of the public health care system.

Demographic features such as rural location, employment and education status were associated with private and public system preferences. Those preferring private care were more likely to endorse living in a metropolitan area and report higher levels of education. For those who chose public care as their preferred option, cost was the most endorsed reason. Concerns around access and affordability of care and treatment corroborate previous findings in Australian bipolar samples and population representative samples (Fisher et al., 2018). More research is needed to determine the impact of economic and educational factors on access to care to address service equity among the Australian population.

Greater awareness and education around bipolar disorder and reducing stigma were seen as key to improving access to mental health care. Several participants reported self-stigma, whilst others described feeling misunderstood, unaccepted, or unsupported by colleagues, health professionals, friends, and family. These experiences are consistent with those described in other reports of international studies of people living with bipolar disorders (Perich et al., 2022). However, a need for greater awareness and education was recognised for the wider community, family, and friends to promote understanding, empathy, and support is needed in Australia, whilst more research is needed to understand the impact of stigma and discrimination for Australians living with bipolar disorder.

Improved training and knowledge in medical and allied health staff was viewed as important for improving illness management and providing patient-centred care. In addition, participants indicated that a change in attitudes towards mental health, along with a systemic change, was needed to improve inpatient care in the future. In line with recovery-orientated approaches, this suggests that participants also prefer to move away from a negative personal identity associated with mental illness (Dell et al., 2021). More work is needed to understand the knowledge needs of those working in healthcare settings who engage with those living with bipolar disorder and to further implement recovery-orientated care in Australia.

Improved inpatient care environments (e.g. less clinical, clean, comfortable) and recovery- oriented approaches to care were identified as unmet needs for inpatient care. This is consistent with research examining patient preferences in selecting hospitals, where respondents valued the hospital appearance and cleanliness, and how quickly they could return to functioning after discharge (Ellis et al., 2020). A desire for more non-health support services and activities (e.g. peer supports, social participation activities) available during inpatient care was also reported.

Participants reported a need for more alternative care needs for crisis care, such as Safe Haven options, a service which include access to peer-support workers in New South Wales. This finding corresponds with prior research that suggests that peer options are a preferred way to engage in early intervention services in Australia (Gates et al., 2024) and suggests that more is needed to ensure that access to peer-support is available consistently to those living with bipolar disorder across other states in Australia outside of New South Wales. This is finding is also consistent with recovery-orientated approaches, which highlight peer-support (Chatwiriyaphong et al., 2024) and further suggests that recovery approaches are a preferred approach for those living with bipolar disorder in Australia.

For treatment setting preferences, people living with bipolar I were less likely to prefer the outpatient hospital setting for early support and treatment. Meanwhile, participants who were significantly younger preferred to receive early mental health support at home, and as outpatients. This may signal a shift in community preferences away from institutional care that parallels the shift in the past 50 years from institutional care to community-based models (Towicz et al., 2021). For those with bipolar I, it may also be that features of their illness are managed differently than those with bipolar II. More research is needed to understand participant experiences of outpatient care specifically in Australia.

### Limitations

There are several limitations to the current study. Firstly, as people self-selected to participate, findings could reflect the perspectives of those with more interest and could differ from perspectives of a community sample. Future research should attempt to explore treatment needs and preferences in a sample that is representative of the general population. Secondly, the sample size was not large enough to conduct some of the analyses. This can be addressed in future studies through larger sample sizes. There are other limitations regarding the use of chi-square in this study where the analyses did not account for confounding variables and no corrections were made for multiple comparisons. Larger studies should further consider the use of analyses that allow for these corrections. There was also a bias towards a female sample, and a low number of participants born outside of Australia took part. More work is needed to engage participants of other gender groups and cultural backgrounds to ensure a greater representation of the broader community.

Future research should also be conducted to explore reasons behind preferred settings for receiving early mental health support. Additionally, the appropriateness of community rehabilitation units for people living with bipolar should be investigated given respondents’ demand for non-institutionalised inpatient care. Lastly, longitudinal studies should be conducted to track changes in consumer preferences alongside changes in healthcare.

## Conclusion

Although recovery-orientated care is highlighted as important in Australian mental health service delivery, several barriers to care and treatment were noted. People living with bipolar disorder reported experiencing unmet treatment and care needs in terms of affordability, access, process needs, awareness, education, stigma, resourcing, and social support. Current services, facilities, and models to delivering care are also perceived to be insufficient for treatment and care needs. More research is needed on understanding the reasons behind preferences for early mental healthcare treatment setting, the suitability and effectiveness of non-institutionalised care, and changes in treatment preferences and needs over time. Government policy should consider improving access to care in Australia consistently, taking into consideration geographical and financial barriers.

## Data Availability

Data is available from the authors upon reasonable request.
